# Delayed wildfires in 2020 promote snowpack melting in the western United States

**DOI:** 10.1073/pnas.2218087120

**Published:** 2023-01-03

**Authors:** Chao You, Chao Xu

**Affiliations:** ^a^College of Environment and Ecology, Chongqing University, Chongqing 400044, China; ^b^CMA Earth System Modeling and Prediction Centre, China Meteorological Administration, Beijing 100081, China; ^c^Institute of Atmospheric Physics, Chinese Academy of Sciences, Beijing 100029, China

Kampf et al. (1) report that the extreme wildfires in 2020 resulted in snow melt occurring approximately 18 d earlier than previously in the late zone and 24 d earlier than previously in the mid-late zone in the western United States. However, the potential impact of changes in wildfire patterns on snowpack melting at high elevations needs to be reconsidered.

**Fig. 1.**
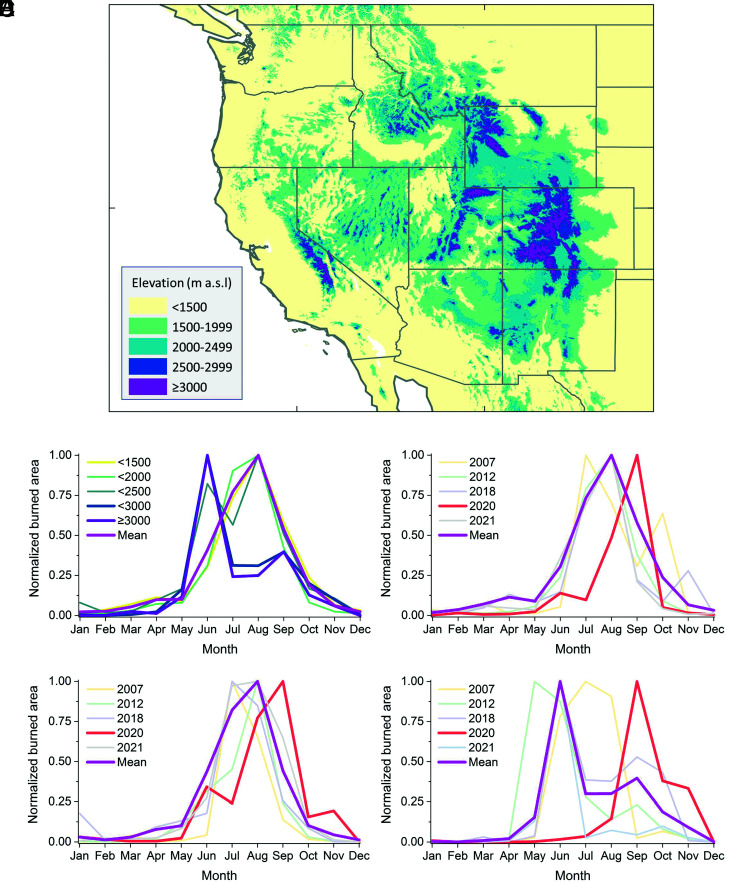
MODIS-derived wildfire patterns in the western United States since 2003. *A*) Elevation categories in the western United States. Panels *B*–*E* show the seasonal variations in wildfires at *B*) different elevation categories; *C*) low elevations (0 to 1,499 m a.s.l.), *D*) middle elevations (1,500 to 2,499 m a.s.l.), and *E*) high elevations (≥2,500 m a.s.l.). Data in panels *B*–*E* were normalized by a min–max method as follows: Xi-XminXmax-Xmin. Elevation data were downloaded from https://www.temis.nl/data/gmted2010/index.php, and MODIS Collection 6 burnt area product was retrieved from https://lpdaac.usgs.gov/products/mcd64a1v006/.

High mountains have experienced more frequent wildfires than previously under climate warming (2–6) and wildfire patterns have also changed (5, 6). The seasonal variations in the extreme wildfires of 2020, particularly in high-elevation regions (Fig. 1*A*), appear to show a lag, relative to other years since 2003, based on the Moderate-Resolution Imaging Spectroradiometer (MODIS) burned area products (Fig. 1 *B*–*E*). This delay of wildfires may have promoted earlier melt of the snowpack in 2021. The late zone, identified by the mean annual snow-free dates in the western United States (1), was included with elevations higher than 2,500 m above sea level (a.s.l.), particularly for regions in the Sierra-Nevada mountains in California and in the southern Rocky Mountains in Wyoming and Colorado (Fig. 1*A*).

For 2003 to 2021, the peak month for wildfires at low elevations (<1,500 m a.s.l.) and intermediate elevations (<2,500 m a.s.l.) is August and advances to June at high elevations (≥2,500 m a.s.l.) in the western United States, based on MODIS-derived observations (Fig. 1*B*). The peak month for wildfires in this period varied between different years for the same elevation category (Fig. 1 *C*–*E*). The five largest wildfire years since 2003 were 2007, 2012, 2018, 2020, and 2021. These years were all characterized by a wildfire-burnt area that was higher than the annual mean burnt area (plus one SD) for all elevations since 2003.

At low and intermediate elevations, the peak month for extreme wildfires varied between July and August but was usually August (Fig. 1 *C* and *D*). In comparison, the peak month for wildfires in 2020 was delayed by about 1 month at low and intermediate elevations. The delay for the timing of the peak in extreme wildfires was clearer at high elevations, where the onset, peak, and end months were around 3 months later in 2020 than the annual mean timings. For example, September was the peak month in 2020, whereas the annual mean peak month, considering all years since 2003, is June (Fig. 1*E*). The extreme wildfires of 2020 extended into November at high elevations, and the area burned in November reached around 430 km^2^, which is more than 100 times the annual mean (calculated for all years since 2003 except 2020).

If wildfires are delayed into winter, impurities such as black carbon and burned woody debris, are likely to be deposited on fresh snow (7). In addition, extreme wildfires can also release large amounts of smoke aerosols, which affect atmospheric conditions and reshape the net radiative on snowpack (8). Since winter snowfall usually begins in October or November in the western United States (7, 9), delayed wildfires may promote earlier onset to snowmelt in the next year.
